# Informing Parents about the Pharmacological and Invasive Behavior Management Techniques Used in Pediatric Dentistry

**DOI:** 10.5681/joddd.2014.017

**Published:** 2014-06-11

**Authors:** Mehrysa Paryab, Hossein Afshar, Razie Mohammadi

**Affiliations:** ^1^Assistant Professor, Department of Pediatric Dentistry, Faculty of Dentistry, Tehran University of Medical Sciences, Tehran, Iran; ^2^Associate Professor, Department of Pediatric Dentistry, Faculty of Dentistry, Tehran University of Medical Sciences, Tehran, Iran; ^3^Private Practice, Department of Pediatric Dentistry, Faculty of Dentistry, Zahedan University of Medical Sciences, Zahedan, Iran

**Keywords:** Child behavior, information, methods, parental consent, pediatric dentistry.

## Abstract

***Background and aims.*** Parental acceptance and consent are important parameters in selecting the required behavior management technique during pediatric dental treatment. The present study sought to assess the effect of three different informing methods on the parental acceptance, consent and concern regarding the pharmacological and invasive behavior management techniques used in pediatric dentistry.

***Materials and methods.*** Ninety mothers of 3-6-year-old uncooperative children were selected and randomly assigned to three study groups. The parents in each group were initially asked to answer three questions related to their levels of ‘acceptance’,‘consent’, and ‘concern’ toward the five behavior management techniques. Then, the information about the techniques was presented through a piece of writing in group I, verbal presentation in group II and showing a film in group III. At last, the parents answered the same three questions again. Score changes were analyzed by using ANOVA, correlations, Mann-Whitney U and Kruskal-Wallis methods.

***Results.*** There were no statistically significant differences in score changes of parental acceptance, consent and concern between the three groups, overall and in relation to each behaviour management technique (P > 0.05). Mothers with aca-demic education revealed more statistically significant concern following presentation of information by film (P < 0.05).

***Conclusion.*** None of the presentation methods had a significant preference over the others; in selecting the behavioral management techniques, it is advisable to observe individual factors, such as the level of education of the mothers.

## Introduction

Pediatric dentistry attempts to create a positive impression in children following a dental visit.^1^ Communicative, advanced and pharmacological techniques have been developed to achieve this purpose.^2^ Moreover; newer techniques are being introduced due to social changes and parental requests.^3^

The American Academy of Pediatric Dentistry (AAPD) holds that an ideal behavior management technique should possess social acceptance along with proper efficiency, and low risk and expenditure.^4^ Professional standards alone may not be sufficient for the acceptance of a behavioral management technique among parents; this means that parents may decide not to accept any of these techniques; even the clinicians think that these are efficient techniques.^5^

Therefore, in communication with the parents, it seems necessary to transfer the information about the pediatric behavior management, and an informed consent has to be obtained as a practical standard for using such techniques during the treatment phase.

The goal of presenting information to the parents is to make them recognize behavior management techniques in children. This may enhance their acceptance and consent and reduce their anxiety in using such techniques.^6^

There is a concern over how the parent should receive the information. In medicine, there have been numerous research studies in colonoscopy, radiotherapy, heart catheterization and rheumatology, as notable examples, on the methods of information presentation and their impact on anxiety and acceptance on the part of patients.^7^ Efforts have also been made in dentistry.^8^ In pediatric dentistry, the amount of acceptability of behavior management techniques and the way of informing the parents about these techniques have attracted the attention of the researchers in this discipline.^6,7,9-14^, Informing the parents about the invasive and pharmacological techniques seems to be more important in clinical applications. The aim of the present study was to assess the impact of various informing methods on the parental acceptance, consent and concern about pharmacological and invasive behavior management techniques used in pediatric dentistry.

## Materials and Methods

The present study was approved by the Ethics Committee and conducted in the Pediatric Clinic of Dental School, in 2011. Ninety parents (mothers) of 3-6-year-old uncooperative children at the first dental visit (behavioral reactions level I & II upon Frankl Behavioral Index) were selected according to the inclusion criteria through convenience sampling. Most of the selected children needed pulp treatment and restoration in at least one of the primary molars. Children with a history of late development, systemic diseases, hospitalization, or a need for emergency dental treatments were excluded from the study. The parents’ native language was Persian, with a minimum high school degree. It was confirmed that the mothers had no experience in using physical restraints and pharmacological techniques during dental treatments of other family members.

The parents were asked to complete a consent form and a questionnaire in order to gather the dental and demographic background data of the child and family. The details of the research were not described in the consent form.

According to balanced block randomization, the parents were then randomly divided into three groups of equal size (N: 30) and surveyed about the five invasive and pharmacological techniques, including physical restraints by dental assistants or parents, physical restraints by special devices (Papoose Board), hand-over-mouth exercise, oral premedication, and general anesthesia. They were initially asked three questions about their acceptance and consent regarding each technique and their concerns about their child’s treatment. The questions had a 5-option Likert type scale format.

1. Do you think this technique is effective? (Acceptance)
from 1: total agreement to 5: total disagreement
2. Would you permit us to use the technique for your kid? (Consent)
from 1: total agreement to 5: total disagreement
3. Are you worried about your child’s treatment? (Concern)
from 1: ‘I am not worried.’ to 5: ‘I am very worried.

Then, the information about the techniques was presented by three different methods based on the group that the parents were included in and at last the parents were asked to answer the same three questions again as follows:

Group I: The parents in this group were given written information on each of the techniques. In each page, one of the techniques was introduced to the parents, followed by the same questions regarding acceptance, consent, and concern about each of the techniques. The information was about the indications of each technique gathered from references of pediatric dentistry.^2^

Group II: The parents in group II were verbally given the same information, and they were subsequently asked to answer the questions of acceptance, consent, and concern. A pediatric dentist who had experience in pediatric dental treatment for about five years performed the presentation of information. The presented information was the same written information presented in the first group.

Group III: The parents in group III were shown a film which included the application of each of the techniques during dental treatments of a 5-year-old uncooperative child with a duration of 30 seconds for each technique. A 15-inch monitor was used to show the film. The produced videotape had been assessed and confirmed by three pediatric dentists. Following the film, the parents were asked to answer the questions related to the same topics of acceptance, consent, and concern again.

The extent of information, the time and the environment for presenting the information in all the three groups were similar. All the information was presented to the parents by one person (who was not the attending dentist). Moreover, each of the parents received the information individually.

After the presentation, the parents immediately received explanations about the reason of the research. None of the parents changed their consent at that time.

The answers before and after presentation were all scored. The differences of the two scores were analyzed using ANOVA, correlation, Mann-Whitney, and Kruskal-Wallis tests. Statistical significance was set at P < 0.05.

## Results

Ninety mothers of 3-6-year-old uncooperative children were enrolled in the study. The mean age was 30 years (age range of 22-42 years). The basic demographic data including the child’s age, kindergarten attendance, the number of the kids in the family, the mothers’ dental experience and level of education were all similar in the three groups.

The percentage of the parents who had chosen choices 1 and 2 of the acceptance question (‘I totally agree’, and ‘I agree’, respectively) revealed that ‘physical restraint by dental assistant or parent’ had the highest acceptance rate, while the two techniques of ‘Papoose Board’ and ‘general anesthesia’ had the lowest rate of acceptance among the parents in all the three groups (Table 1).

**Table 1:** Frequency distribution of parents’ responses to the choices 1 & 2 (‘I totally agree’, and ‘I agree’) for the question related to the acceptance of behavior management techniques

Behavior management techniques	Percent
Physical restraint by dental assistant or mother	82.2 %
Physical restraint by special devices	37.8 %
Hand over mouth	53.3 %
Oral pre-medication	54.4 %
General anesthesia	38.9 %

The comparison of the changes in the scores obtained about the parental acceptance, consent and concern showed no statistically significant differences among the three methods (P > 0.05; Table 2).

**Table 2:** Comparison of the score changes in the parental acceptance, consent and concern among the three methods analyzed by ANOVA

Score change category	Group I	Group II	Group III	P
	Mean (±SD)	Mean (±SD)	Mean (±SD)	
Parental acceptance	-0.60(±3.60)	-0.30(±3.33)	0.27(±4.41)	0.67
Parental consent	0.53(±3.81)	0.03(±2.72)	0.50(±4.67)	0.85
Parental concern	0.70(±5.90)	0.43(±3.33)	1.37(±4.44)	0.73
Group I: parents who obtained written Information				
Group II: parents who obtained oral Information				
Group III: parents who obtained Information through a Film				

The changes in the scores obtained for each of the behavior management techniques were separately studied as well. No significant differences were found in this respect either (P > 0.05) (Table 3).

**Table 3:** Comparison of the score changes in the parental acceptance, consent, and concern among three methods for each behavior management technique by ANOVA

Score change category	Group I	Group II	Group III	P
	Mean (±SD)	Mean (±SD)	Mean (±SD)	
Score changes in acceptance for PR	-0.23(±1.33)	-.016(±1.08)	-.016(±1.91)	0.98
Score changes in consent for PR	-0.20(±1.29)	-0.50(±0.82)	0.10(±1.88)	0.25
Score changes in concern for PR	-0.16(±1.39)	-0.30(±1.05)	-0.63(±1.49)	0.37
Score changes in acceptance for PB	0.23(±1.27)	0.30(±1.36)	0.63(±2.34)	0.63
Score changes in consent for PB	0.60(±1.45)	0.20(±0.96)	0.46(±2.22)	0.62
Score changes in concern for PB	-0.10(±1.44)	-0.16(±1.39)	0.23(±1.61)	0.53
Score changes in acceptance for HOME	-0.50(±1.27)	-0.46(±1.27)	0.13(±1.69)	0.16
Score changes in consent for HOME	-0.06(±1.33)	-0.06(±1.20)	0.23(±1.77)	0.65
Score changes in concern for HOME	-0.33(±1.58)	0.03(±1.21)	-0.23(±1.59)	0.61
Score changes in acceptance for OP	0.00(±1.43)	0.50(±1.13)	0.20(±0.99)	0.27
Score changes in consent for OP	0.2(±1.44)	0.70(±0.95)	0.23(±0.93)	0.17
Score changes in concern for OP	-0.16(±1.70)	0.03(±1.09)	-0.46(±1.40)	0.40
Score changes in acceptance for GA	-0.10(±1.32)	-0.46(±1.16)	-0.53(±1.33)	0.37
Score changes in consent for GA	0.00(±1.20)	-0.30(±1.29)	-0.53(±1.35)	0.27
Score changes in concern for GA	0.06(±1.52)	-0.03(±0.71)	-0.26(±1.17)	0.53
Group I: parents who obtained written Information; Group II: parents who obtained oral Information; Group III: parents who obtained Information through a film; PR: physical restraint by dental assistant				
PB: Papoose board; HOME: Hand over Mouth Exercise; OP: Oral premedication; GA: General anesthesia.				

The correlation coefficients and the Kruskal-Wallis analyses showed that based on the factors of the mothers’ age and dental experience, no statistically significant differences existed in score changes for acceptance, consent and concern between the three experimental groups (P > 0.05). In other words, age and mothers’ previous dental experience had no impact on their acceptance, consent, and concern.

As for the mothers’ level of education, and in score changes for their acceptance and consent, no statistically significant differences were found in mothers who had academic education and those who did not (P > 0.05). However, for the concern of the parents about the treatment of their kids, a significant change was found in Group III for academic education (P = 0.034). In other words, those with a higher level of education expressed more signs of concern following watching the film.

Moreover, a direct correlation was observed in the degree of changes in the scores for ‘acceptance’ and the changes in the scores of their ‘consent’ for performing behavior management techniques, while a reverse correlation was seen between the changes in the scores for ‘acceptance’ and those in their level of ‘concern’ (P ≤ 0.0001). In other words, the more ‘acceptance’ level of a parent, the more ‘consent’ they would have, and hence, the less their level of ‘concern’ would be for the behavior management techniques used.

## Discussion

In some children, due to low chronological or cognitive age, or due to some emotional problems, the need for using some pharmacological and invasive behavior management techniques increases. In most of such studies, the pharmacological and invasive methods have lower rates of acceptance;^6,10-23^ However, their attitude may positively change in the long term after the use of these techniques.^24^ Therefore, it seems that parents’ knowledge and acceptance in using pharmacological and invasive methods are highly necessary, especially during first sessions.

The results about the acceptability of different behavior management techniques showed that regardless of the ways the information was presented, the technique of ‘physical restraint using special devices’ had the lowest acceptance rate among parents, which is in line with findings of other studies.^6,10-20^ For the two techniques of ‘general anesthesia’ and ‘hand over mouth’, the mothers preferred the latter one. The low acceptance rate in using ‘general anesthesia’ was observed among all the mothers with different ages, education, and previous dental experiences; and uncooperativeness of their children could not increase their acceptance rate, either. This might be due to the fact that the children in our study had no emergency dental treatment needs. The more serious the treatment, the more the parents will accept the more invasive treatment protocols.^15,17^ Most Iranian parents think that the general anesthesia process may negatively affect the brain function and intelligence of the child. Therefore, in children with no emergency treatment needs, the parents prefer to use other techniques. Havelka et al^14^ and Scott and Garcia-Godoy^11^ have shown higher rates of acceptance for the technique of ‘general anesthesia’ among parents with a lower social status, and they concluded that the social and educational levels of the mothers, as well as their previous information about the risk factors inherent in ‘general anesthesia’ had been the important and effective elements in their acceptance and consent rates.

The type of treatment needed,^15,17^ parents’ previous dental experiences,^21,22^ parents’ personality traits,^23^ and parents’ anxiety and their socio-economic statuses^11,12,14^ have all been among the important factors which would have serious impacts on parental consent in using behavior management techniques for the treatment of their children.

The way and the amount of information presented^7^ and who offers the information to parents^13^are other important effective factors. In the past and as a routine, the necessary information has been given verbally.^7^ Direct patient/physician relationship is the basis of such an approach. However, due to the different abilities physicians have, it has been suggested to use other on-the-side forms of presentation of information along with the verbal forms. One group of such methods is the use of leaflets. Some parents do not study them. Others may not perceive what is said in them. Today, the tendency towards electronic audio-visual methods has increased. The use of a film will save time for the dentist. There is concern that the details visible in the film may increase anxiety among the parents and reduce their rate of acceptance, or even make them avoid continuation of the treatment of their children.^7^

Due to the importance of acceptance and consent of parents about the pharmacological and invasive techniques, a film about the application of these techniques was produced and shown to the mothers of uncooperative children.

The results about the effectiveness of different informing methods showed that leaflets would be less effective in increasing acceptance and consent of the mothers, though the results had not been significant. The verbal approach of informing the parents may be efficient more than the written information. Allen et al reached the same conclusions in their study.^6^ The positive effect of verbal presentation of information has been noted, especially among parents with higher socio-economic status.^9,10,12,14^

The results of this study showed that mothers with a higher level of education showed more signs of concern following watching the film. The produced film did not have any verbal explanation. Therefore, it seems that showing a film with the details of pharmacological and invasive techniques may increase parents’ concerns about their child’s treatment, especially in those with higher academic education. Even if the positive effects and success of such techniques as ‘physical restraint with special devices’ or ‘hand over mouth’ are presented in a film, it should be kept in mind that showing a film alone cannot be an alternative approach. Lawrence et al^10^ and Abushal ^12^ noted that verbal presentation of information along with showing a film would be effective in order to increase the acceptance rates for using invasive techniques, such as ‘physical restraint’ and ‘hand over mouth’.

It is suggested that in future studies on the methods for presenting information, the effects of personality and the socio-economic statuses of the parents be investigated more carefully.

## Conclusion

1. None of the methods for presenting information had any preference over the other in behavior management, though it seems that verbal presentation has to be performed by the dental team alongside the other forms in presenting information.
2. In deciding for a method to present information, individual factors, such as the educational level of the mothers, have to be considered.
